# Supplementary material for: Strategies for feeding the world more sustainably with organic agriculture
Source: Nat Commun. 2017 Nov 14;8:1290. doi: 10.1038/s41467-017-01410-w (PMC5686079; doi:10.1038/s41467-017-01410-w)
Supplement: Supplementary file 2 — Supplementary Information [file 41467_2017_1410_MOESM2_ESM.pdf]

Supplementary Table 1: Comparison of aggregate N quantity categories for SOLm and Tubiello 2013[68]

N (megatons)	SOLm	Tubiello 2013[68]
N in manure (total in all manure management systems incl. left on pastures)	115	113
N in manure applied to croplands	20	25
N in manure applied to grasslands	48	40*
N from mineral fertilizers	98	100
N in total crop residues	34	30
N fixation in crops (without rice and sugar cane)	25	22**

*equals the total N quantity in manure left on pastures minus the N volatized and leached from N in manure left on pastures
**the source for this number is not Tubiello (2013)[68] but Herridge et al. (2008)[47].

Supplementary Table 2: Comparison of aggregate GHG emission categories for SOLm, Tubiello 2013 and Gerber et al. 2013[68,69]

GHG emissions (gigatons)	SOLm	Tubiello 2013[68]	Gerber et al. 2013[69]
Methane from rice production	0.49	0.5	
Enteric fermentation	2.32	2.02	2.78
Manure management CH4	0.31	0.22	0.31
Manure management N2O total	0.31	0.12	0.37
Manure management N2O direct	0.11	0.11	
Manure management N2O indirect	0.2	0.01	
Synthetic fertilizer application (direct plus indirect)	0.58	0.65	
Crop residues application	0.16	0.18	
Manure application (on crops and grass)	0.82	0.97	1.16

Supplementary Table 3: Pesticide model classifications

Rating	Pesticide level per crop (PUI)
0	No harmful pesticides* used
1	Low level of pesticide application
2	Medium level of pesticide application
3	High level of pesticide application / harmful pesticides used*
	* WHO classification

Rating	Pesticide legislation per country (PL)
0	All chem.-synthetic pesticides (WHO-classes 1-2) banned
1	Rigid pesticide legislation and control excludes harmful pesticides*
2	Average pesticide legislation and control
3	Legislation does not preclude the use of harmful pesticides*
	*WHO classification

Rating	Access to pesticides per country (AP)
0	Farmers have no access to chem.-synthetic (WHO-classes 1-2) pesticides
1	Only few farmers have access to chem.-synthetic pesticides (max. 10% of the cultivated land is treated with pesticides)
2	Some farmers have access to chem.-synthetic pesticides (10-50% of the land that deserves treatment is treated)
3	Many farmers have access to chem.-synthetic pesticides (min. 50% of the land that deserves treatment is treated)

Supplementary Table 4: Country-specific ratings of pesticide legislation (PL) and the accessibility of pesticides to farmers (AP)

Country	PL	AP
Afghanistan	3	1
Albania	2.5	2.5
Algeria	2	2
American Samoa	2	3
Andorra	2	3
Angola	3	1
Anguilla	2	3
Antigua and Barbuda	2	3
Argentina	2	2.5
Armenia	3	3
Aruba	2	3
Australia	1	3
Austria	1	3
Azerbaijan	3	2
Bahamas	2	3
Bahrain	2	2.5
Bangladesh	3	1
Barbados	2	3
Belarus	2.5	3
Belgium	1	3
Belize	2	3
Benin	2.5	1
Bermuda	2	3
Bhutan	3	1.5
Boli*via* (Plurinational State of)	3	2
Bosnia and Herzegovina	2	3
Botswana	2	1.5
Brazil	1.5	2.5
British Virgin Islands	2	3
Brunei Darussalam	2	2
Bulgaria	2.5	3
Burkina Faso	2.5	1
Burundi	2	2
Cambodia	3	1
Cameroon	3	2
Canada	1	3
Cape Verde	3	1.5
Cayman Islands	2	3
Central African Republic	3	1
Chad	3	1
Channel Islands	1	3
Chile	2	3
China	3	2
Colombia	2	2.5
Comoros	2	2
Congo	3	1
Cook Islands	1.5	3
Costa Rica	2	3
Côte d'Ivoire	3	1
Croatia	1.5	3
Cuba	2.5	2
Cyprus	2	3
Czech Republic	2	3
Democratic People's Republic of Korea	3	1.5
Democratic Republic of the Congo	3	1
Denmark	1	3
Djibouti	3	2
Dominica	2	2.5
Dominican Republic	2	2.5
Ecuador	2.5	2.5
Egypt	2	3
El Salvador	2	3
Equatorial Guinea	3	2
Eritrea	2.5	1
Estonia	1.5	3
Ethiopia	2	1.5
Falkland Islands (Malvinas)	2	3
Faroe Islands	1	3
Fiji	2	3
Finland	1	3
France	1	3
French Guiana	2	2
French Polynesia	1.5	3
Gabon	3	1.5
Gambia	2.5	1
Georgia	3	2
Germany	1	3
Ghana	2	1.5
Gibraltar	2	3
Greece	2	3
Greenland	1	2.5
Grenada	2	3
Guadeloupe	2	2.5
Guam	2	3
Guatemala	2	3
Guinea	2.5	1
Guinea-Bissau	3	1
Guyana	2.5	2
Haiti	3	2
Honduras	2	3
Hungary	2	3
Iceland	1	3
India	3	1.5
Indonesia	3	1
Iran (Islamic Republic of)	3	2
Iraq	2	2
Ireland	1	3
Isle of Man	1	3
Israel	2	3
Italy	1.5	3
Jamaica	2	2.5
Japan	1	3
Jordan	2	3
Kazakhstan	3	2
Kenya	2	2
Kiribati	2	3
Kuwait	2	3
Kyrgyzstan	3	2
Lao People's Democratic Republic	3	1
Lat*via*	1.5	3
Lebanon	2	2
Lesotho	2	2
Liberia	3	1
Libya	2	2
Liechtenstein	2	3
Lithuania	1.5	3
Luxembourg	1	3
Madagascar	2	2
Malawi	2	2
Malaysia	2	1.5
Maldives	2	2
Mali	2.5	1
Malta	1.5	3
Marshall Islands		
Martinique	2	3
Mauritania	2.5	1
Mauritius	2	2
Mayotte	3	1
Mexico	2	2.5
Micronesia (Federated States of)	2	3
Mongolia	3	1
Montenegro	2	3
Montserrat	2	3
Morocco	1	2
Mozambique	2	2
Myanmar	3	1
Namibia	2	2
Nauru	2	3
Nepal	3	1
Netherlands	1	3
Netherlands Antilles	2	3
New Caledonia	1.5	3
New Zealand	1	3
Nicaragua	2	2.5
Niger	2.5	1
Nigeria	3	1
Niue	2	2.5
Norfolk Island		
Northern Mariana Islands		
Norway	1	3
Occupied Palestinian Territory	3	1
Oman	2	3
Pakistan	3	1
Palau		
Panama	2	3
Papua New Guinea	2.5	1.5
Paraguay	2	3
Peru	2.5	2
Philippines	2.5	1.5
Pitcairn Islands		
Poland	2	3
Portugal	1	3
Puerto Rico	2	2
Qatar	2	3
Republic of Korea	1.5	3
Republic of Moldova	3	2.5
Réunion	3	2
Romania	2	3
Russian Federation	3	2
Rwanda	2	2
Saint Helena	3	1
Saint Kitts and Nevis	2	3
Saint Lucia	2	3
Saint Pierre and Miquelon	1	3
Saint Vincent and the Grenadines	2	3
Samoa	2	3
San Marino	2	3
Sao Tome and Principe	3	2
Saudi Arabia	2	3
Senegal	2	1
Serbia	2	3
Serbia and Montenegro	2	3
Seychelles	3	2
Sierra Leone	3	1
Singapore	2.5	2.5
Slovakia	2	3
Slovenia	2	3
Solomon Islands	2	3
Somalia	3	1
South Africa	2	2
Spain	1.5	3
Sri Lanka	2.5	1
Sudan	3	1
Suriname	2.5	2
Swaziland	3	2
Sweden	1	3
Switzerland	1	3
Syrian Arab Republic	2	2
Tajikistan	3	2
Thailand	2.5	1
The former Yugoslav Republic of Macedonia	2	3
Timor-Leste	3	1
Togo	2.5	1
Tokelau	2	2.5
Tonga	2	3
Trinidad and Tobago	2	3
Tunisia	2	2
Turkey	2	2
Turkmenistan	3	2.5
Turks and Caicos Islands	2	3
Tuvalu	2	2.5
Uganda	2	2
Ukraine	2.5	3
United Arab Emirates	2	3
United Kingdom	1	3
United Republic of Tanzania	2	2
United States of America	1	3
United States Virgin Islands	1.5	3
Uruguay	2	3
Uzbekistan	3	2
Vanuatu	2	3
Venezuela (Bolivarian Republic of)	2	2.5
Viet Nam	3	1
Wallis and Futuna Islands	2	3
Western Sahara	2	1
Yemen	2	2
Yugoslav SFR	2	3
Zambia	3	1.5
Zimbabwe	3	1

PL = Pesticide legislation; AP = accessibility of pesticides to farmers

Supplementary Table 5: Crop-specific pesticide use intensity (PUI)

Activity	PUI
Agave Fibres Nes	1
Alfalfa For Forage+Silag	0
Alfalfa Meal And Pellets	0
Almonds, with shell	2
Anise, badian, fennel, corian.	1
Apples	3
Apricots	3
Arabic Gum	0
Arecanuts	1
Artichokes	2.5
Asparagus	2.5
Avocados	3
Bambara beans	2
Bananas	3
Barley	2
Beans, dry	3
Beans, green	2
Beets For Fodder	0
Berries Nes	2
Blueberries	2
Brazil nuts, with shell	1
Broad beans, horse beans, dry	3
Buckwheat	1
Cabbages and other brassicas	3
Canary seed	1
Carobs	2
Carrots and turnips	3
Cashew nuts, with shell	1.5
Cashewapple	2
Cassava	2.5
Castor oil seed	3
Cauliflowers and broccoli	3
Cereals, most	0
Cereals, nes	2
Cherries	3
Chestnuts	1.5
Chick peas	3
Chicory roots	2.5
Chillies and peppers, dry	2.5
Chillies and peppers, green	2.5
Cinnamon (canella)	2
Citrus fruit, nes	3
Clover For Forage+Silage	0
Cloves	3
Cocoa beans	3
Coconuts	2.5
Coffee, green	3
Coir	2
Cow peas, dry	3
Cranberries	1
Cucumbers and gherkins	3
Currants	2
Dates	3
Eggplants (aubergines)	3
Eggs Excl Hen	0
Fibre Crops Nes	3
Figs	2
Flax fibre and tow	2
Fonio	1
Forage Products Nes	0
Fruit Fresh Nes	3
Fruit, tropical fresh nes	3
Fruits, most	0
Garlic	1.5
Ginger	2
Gooseberries	3
Grapefruit (inc. pomelos)	3
Grapes	3
Grass	0
Grasses Nes,Forage+Silag	0
Groundnuts, with shell	2
Gums Natural	3
Hay (Clover,Lucerne,Etc)	0
Hay (Unspecified)	0
Hay Non-Leguminous	0
Hazelnuts, with shell	1
Hemp Tow Waste	2
Hempseed	1
Hops	2
Jojoba	0
Jute	2
Kapok Fruit	2
Karite Nuts (Sheanuts)	1
Kiwi fruit	3
Kolanuts	1
Leguminous Nes,For+Sil	0
Leguminous vegetables, nes	2.5
Lemons and limes	3
Lentils	1
Lettuce and chicory	2.5
Linseed	1
Lupins	1
Maize	3
Maize For Forage+Silage	0
Maize, green	3
Mangoes, mangosteens, guavas	3
Manila Fibre (Abaca)	2
Maple	0
Maté	2
Melonseed	1
Millet	2.5
Mixed grain	2
Mules	0
Mushrooms and truffles	0
Mustard seed	1
Natural rubber	3
Nutmeg, mace and cardamoms	2
Nuts, nes	1.5
Oats	1
Oil Of Citronella	0
Oil palm fruit	3
Oils Marine Animals	0
Oilseeds, Nes	2.5
Okra	2
Olives	2
Onions (inc. shallots), green	2
Onions, dry	2
Oranges	3
Other Bastfibres	2
Other melons (inc.cantaloupes)	2.5
Papayas	3
Peaches and nectarines	3
Pears	3
Peas, dry	3
Peas, green	3
Pepper (Piper spp.)	2
Peppermint	1
Persimmons	2
Pigeon peas	2
Pineapples	2.5
Pistachios	1
Plantains	3
Plums and sloes	3
Pome Fruit Nes	3
Popcorn	2.5
Poppy seed	1
Potatoes	3
Pulses, nes	2
Pumpkins, squash and gourds	3
Pyrethrum,Dried	0
Quinces	3
Quinoa	1
Ramie	1
Rapeseed	3
Raspberries	3
Rice, paddy	3
Roots and Tubers, most	0
Roots and Tubers, nes	2.5
Rye	1
Safflower seed	2
Seed cotton	3
Sesame seed	2
Sisal	2
Sorghum	2.5
Sour cherries	3
Soybeans	3
Spices, nes	2
Spinach	3
Starch and Sugar crops for Alc	0
Stone fruit, nes	3
Straw, Husks	0
Strawberries	3
String beans	3
Sugar beet	2.5
Sugar cane	3
Sugar crops, nes	2.5
Sunflower seed	1
Swedes For Fodder	0
Sweet potatoes	2.5
Tallow tree	0
Tangerines, mandarins, clem.	3
Taro (cocoyam)	2.5
Tea	3
Tea Nes	0
Tobacco, unmanufactured	3
Tomatoes	3
Triticale	2
Tung Nuts	1
Turkeys	0
Turnips For Fodder	0
Vanilla	2
Vegetables fresh nes	2.5
Vegetables, most	0
Vegetables+Roots,Fodder	0
Vetches	1
Walnuts, with shell	1.5
Watermelons	3
Wheat	2
Yams	2.5
Yautia (cocoyam)	2.5

PUI = Pesticide use intensity; Nes = other, not elsewhere specified

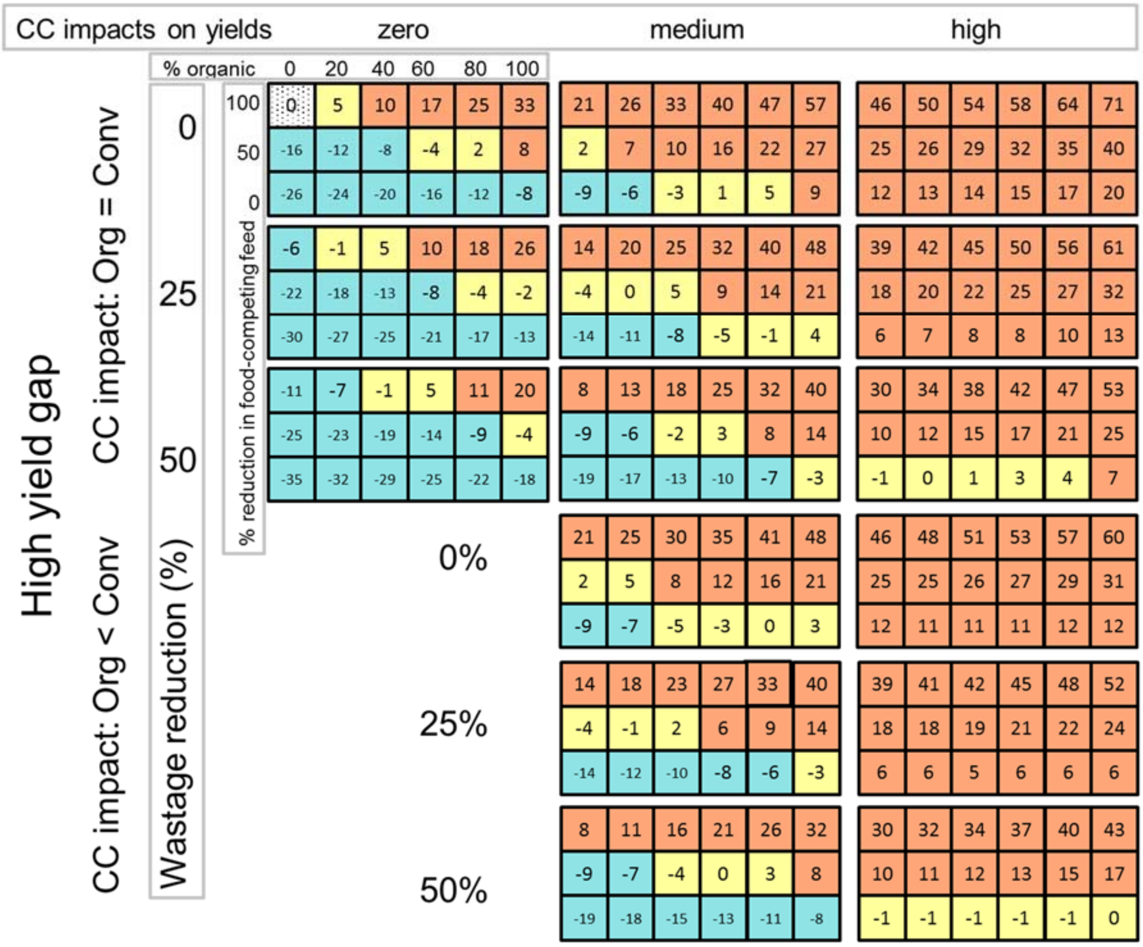

Supplementary Figure 1: Cropland area change with high yield gaps assumed

Percentage change in cropland areas with respect to the reference scenario (0% organic agriculture, no changes in livestock feed and food waste). Calories are kept constant for all scenarios. High yield gaps[10]; scenarios differ in organic shares (0-100%); impacts of climate change on yields (low, medium, high; lower impacts for organic than conventional agriculture); food-competing feed reductions (0, 50, 100% reduced from the levels in the reference scenario) and wastage reduction (0,25,50% with respect to the reference scenario). Colour code for the relation to reference scenario value that is displayed in dotted grey: >+5%: red, <-5% blue, between -5% and +5% yellow; in the reference scenario, cropland areas are 6% higher than in the baseline today.

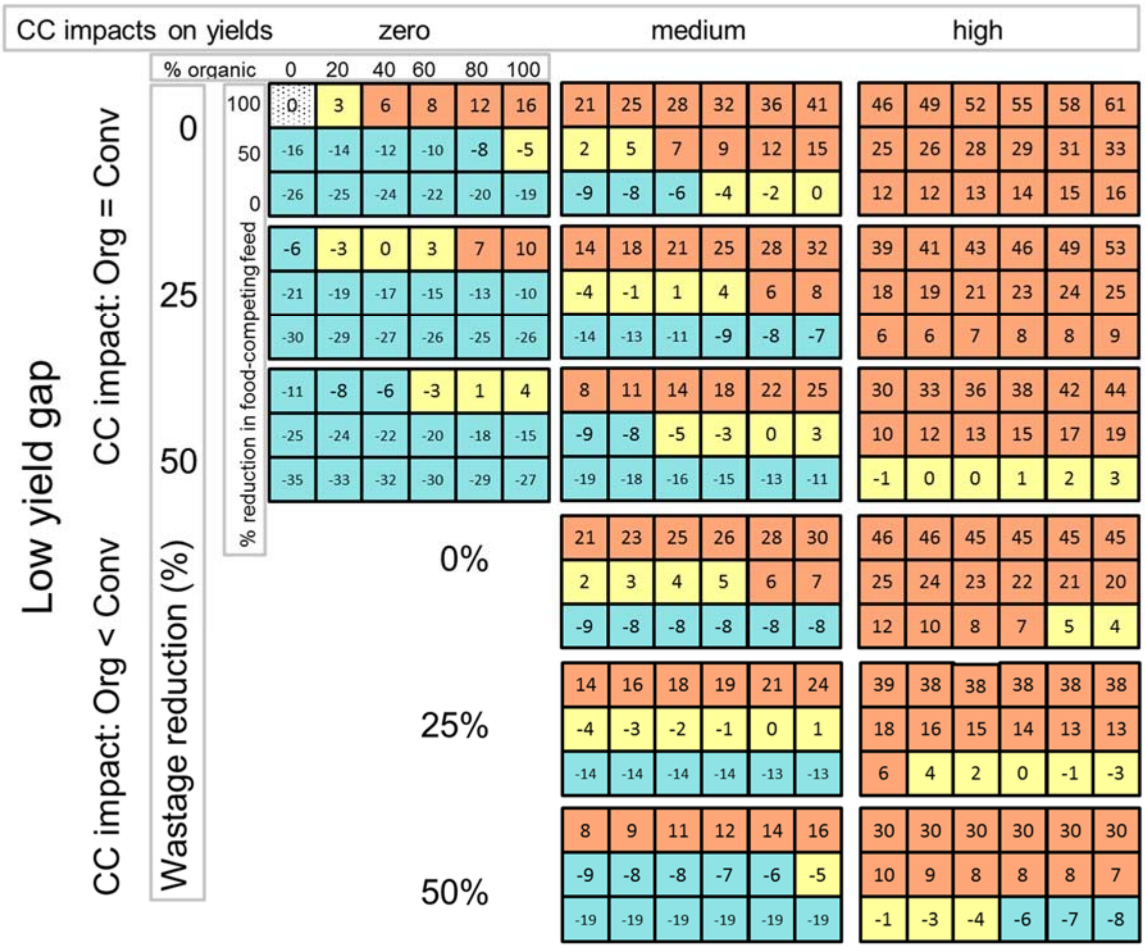

Supplementary Figure 2: Cropland area change with low yield gaps assumed

Percentage change in cropland areas with respect to the reference scenario (0% organic agriculture, no changes in livestock feed and food waste). Calories are kept constant for all scenarios. Low yield gaps[9]; scenarios differ in organic shares (0-100%); impacts of climate change on yields (low, medium, high; lower impacts for organic than conventional agriculture); food-competing feed reductions (0, 50, 100% reduced from the levels in the reference scenario) and wastage reduction (0,25,50% with respect to the reference scenario). Colour code for the relation to reference scenario value that is displayed in dotted grey: >+5%: red, <-5% blue, between -5% and +5% yellow; in the reference scenario, cropland areas are 6% higher than in the baseline today.

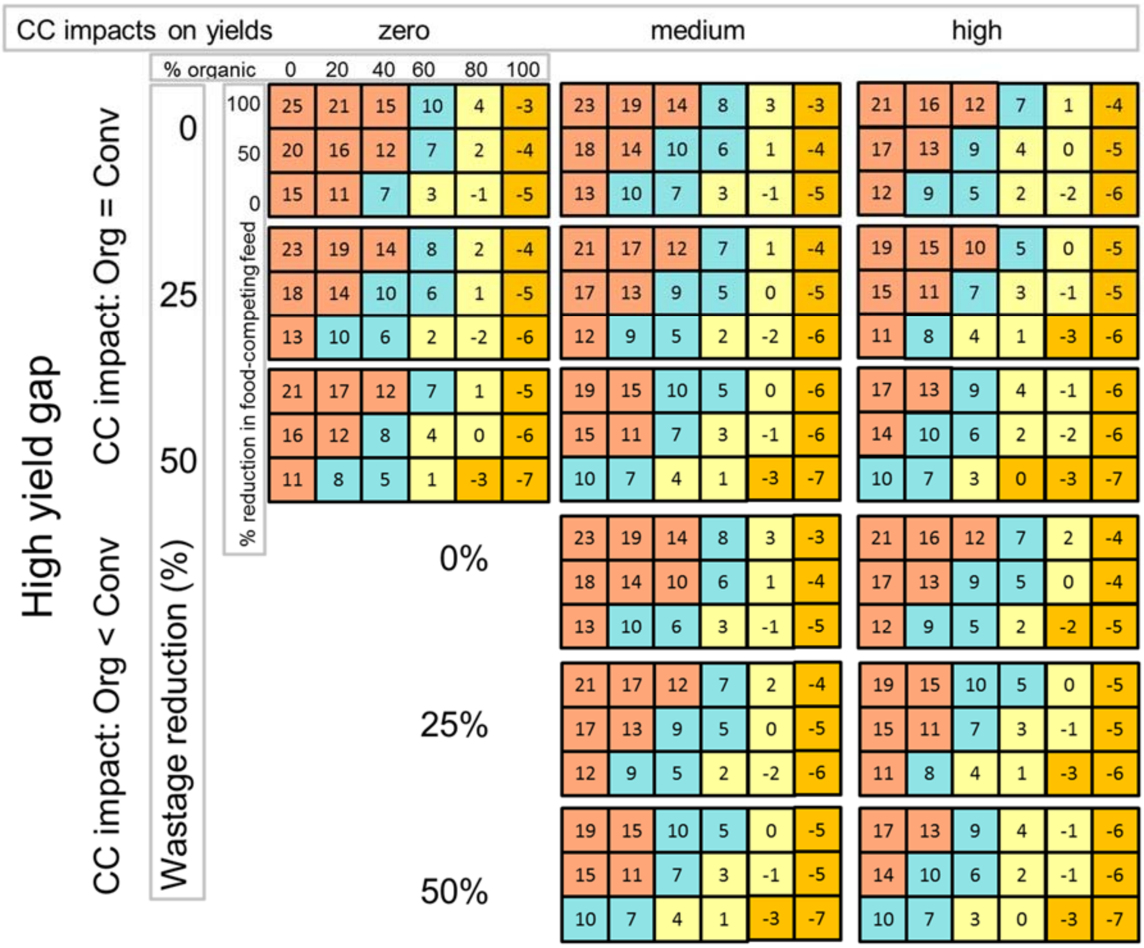

Supplementary Figure 3: Nitrogen balance with high yield gaps assumed

N-surplus (positive values) or deficit (negative values) in kg N/ha. Calories are kept constant for all scenarios. High yield gaps[10]; scenarios differ in organic shares (0-100%); climate change impacts (low, medium, high; lower impacts for organic than conventional agriculture); food-competing feed reductions (0, 50, 100% reduced from the levels in the reference scenario) and wastage reduction (0,25,50% with respect to the reference scenario). Colour code for the relation to reference scenario values (0% organic agriculture, no changes in livestock feed and food waste): >10kg/ha: red (unsustainably high), between 10kg/ha and 5kg/ha blue (optimum, reduction from current average surplus by 60-80%[31,32]), between 4kg/ha and -2kg/ha yellow (critical, rather low), <-2kg/ha red (deficit).

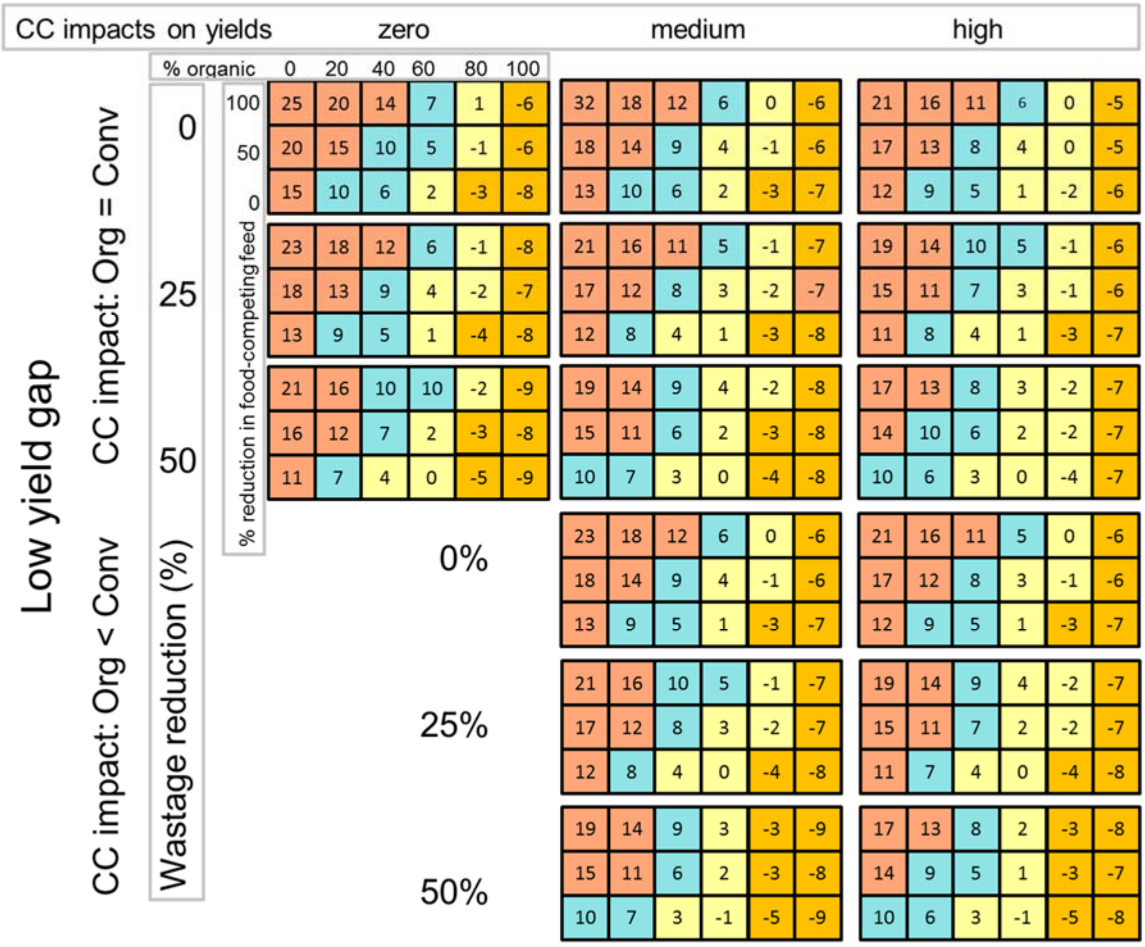

Supplementary Figure 4: Nitrogen balance with low yield gaps assumed

N-surplus (positive values) or deficit (negative values) in kg N/ha. Calories are kept constant for all scenarios. Low yield gaps[9]; scenarios differ in organic shares (0-100%); climate change impacts (low, medium, high; lower impacts for organic than conventional agriculture); food-competing feed reductions (0, 50, 100% reduced from the levels in the reference scenario) and wastage reduction (0,25,50% with respect to the reference scenario). Colour code for the relation to reference scenario values (0% organic agriculture, no changes in livestock feed and food waste): >10kg/ha: red (unsustainably high), between 10kg/ha and 5kg/ha blue (optimum, reduction from current average surplus by 60-80%[31,32]), between 4kg/ha and -2kg/ha yellow (critical, rather low), <-2kg/ha red (deficit).

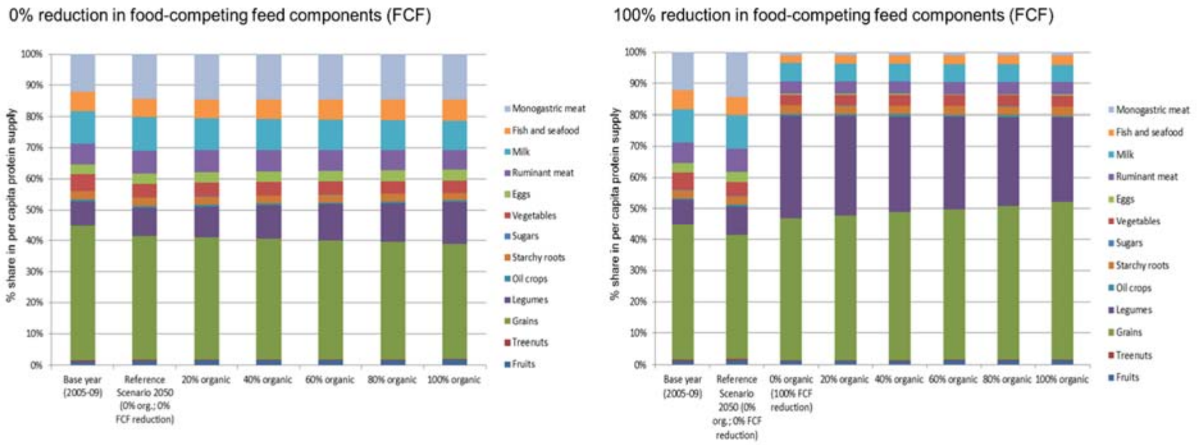

Supplementary Figure 5: Dietary composition under high yield gaps and without impact of climate change on yields

Dietary composition as shares of various commodity groups in per capita protein supply (before subtracting wastage) without (left) and with (right) reduction of food-competing feed; no climate change impacts; high yield gaps[10]. All scenarios provide the same amount of calories (no wastage reduction).

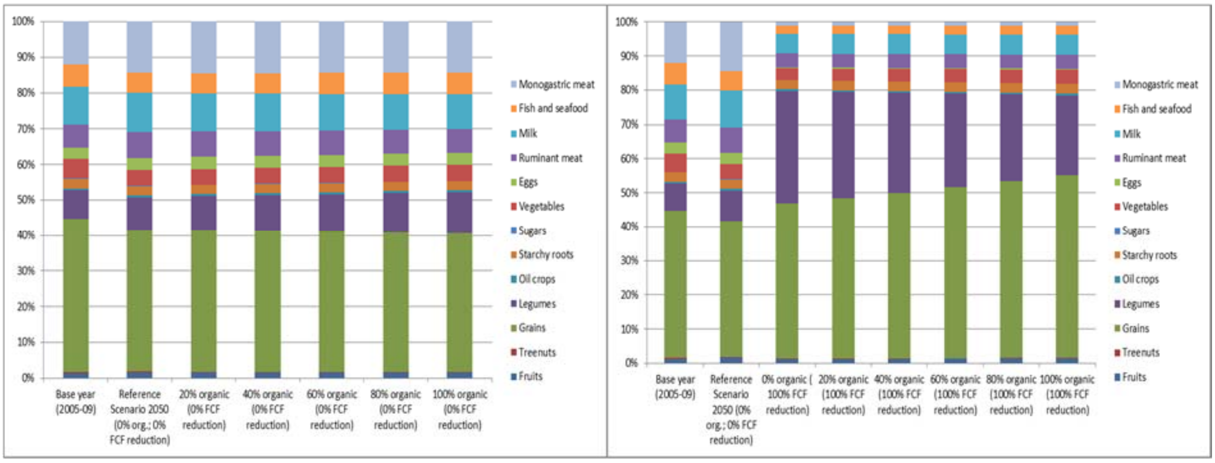

Supplementary Figure 6: Dietary composition under low yield gaps and without impact of climate change on yields

Dietary composition as shares of various commodity groups in per capita protein supply (before subtracting wastage) without (left) and with (right) reduction of food-competing feed; no climate change impacts; low yield gaps[9]. All scenarios provide the same amount of calories (no wastage reduction).

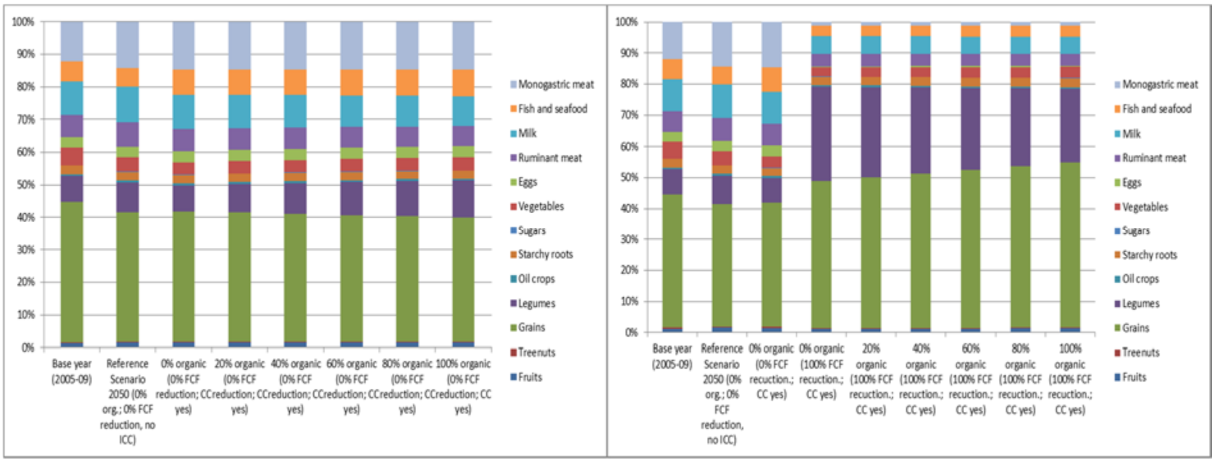

Supplementary Figure 7: Dietary composition under high yield gaps and with impact of climate change on yields

Dietary composition as shares of various commodity groups in per capita protein supply (before subtracting wastage) without (left) and with (right) reduction of food-competing feed; high climate change impacts; high yield gaps[10]. All scenarios provide the same amount of calories (no wastage reduction).

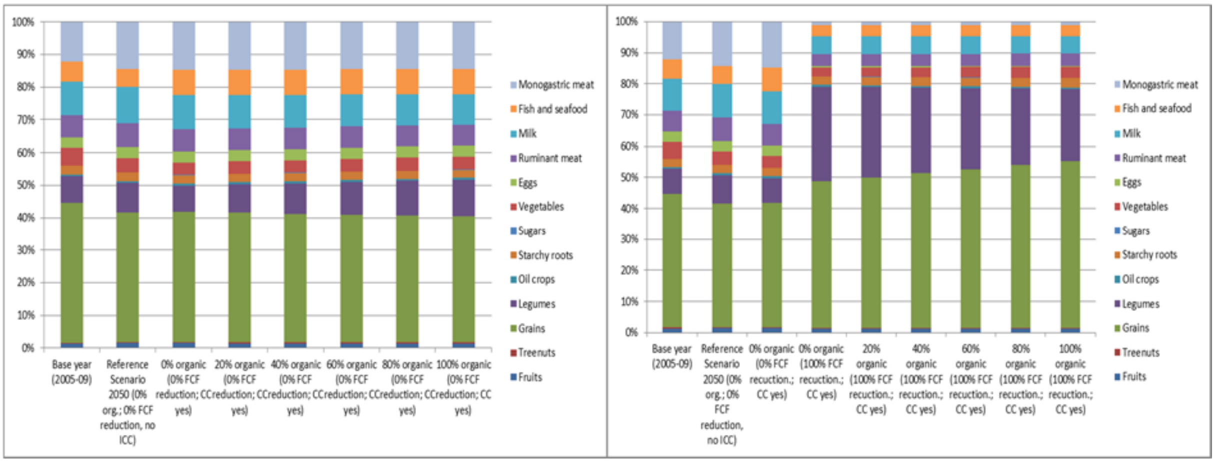

Supplementary Figure 8: Dietary composition under low yield gaps and with impact of climate change on yields

Dietary composition as shares of various commodity groups in per capita protein supply (before subtracting wastage) without (left) and with (right) reduction of food-competing feed; high climate change impacts; low yield gaps[9]. All scenarios provide the same amount of calories (no wastage reduction).

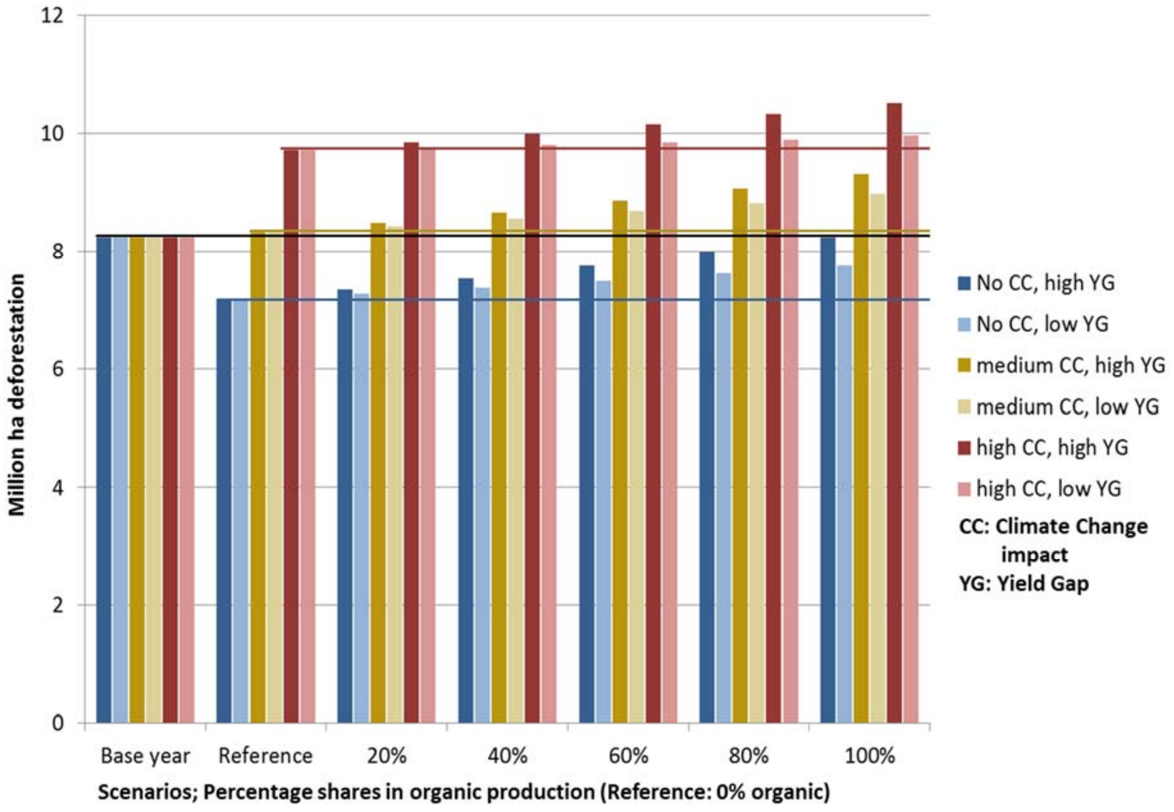

Supplementary Figure 9: Deforestation

Deforestation (million ha) for the base year, the reference scenarios (0% organic) and scenarios with increasing percentages of organic production. Displays scenarios with low and high yield gaps[9,10] without and with medium and full impacts of climate change on yields (no / medium / high CC).

0% food wastage reduction

0% reduction in food-competing feed (FCF)

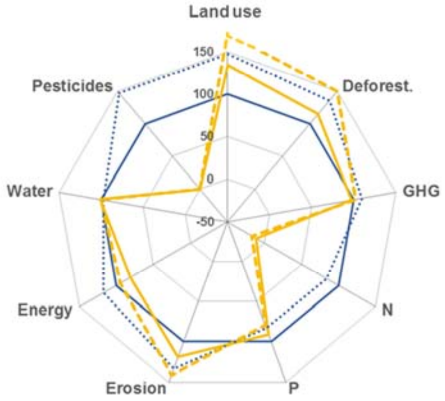

50% reduction in FCF

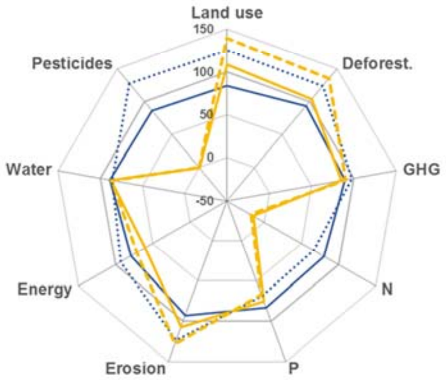

100% reduction in FCF

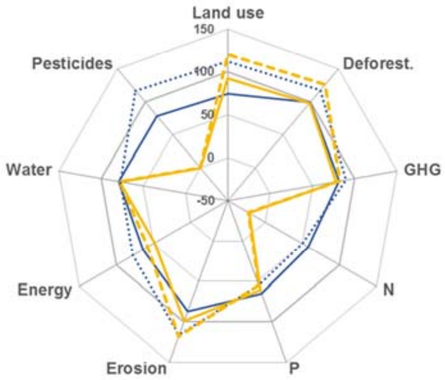

The numbers on the axis indicate % impact, rel. to the reference scenario (which is represented by the thicker grey line at 100%)

25% food wastage reduction

0% reduction in food-competing feed (FCF)

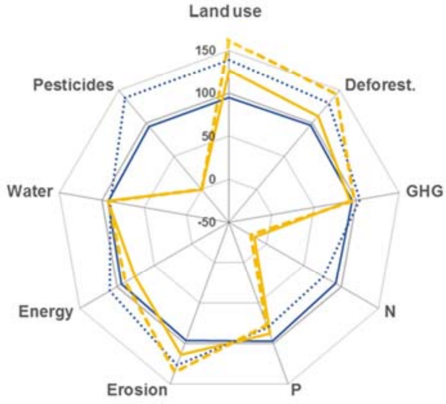

50% reduction in FCF

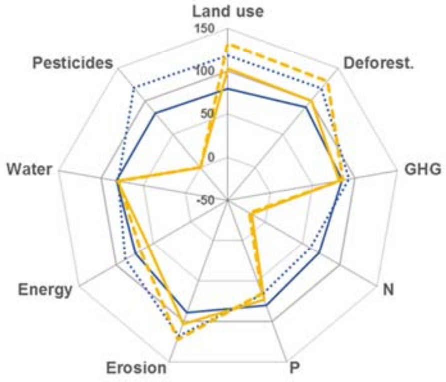

100% reduction in FCF

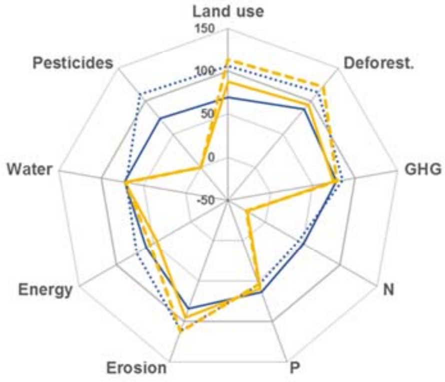

The numbers on the axis indicate % impact, rel. to the reference scenario (which is represented by the thicker grey line at 100%)

50% food wastage reduction

0% reduction in food-competing feed (FCF)

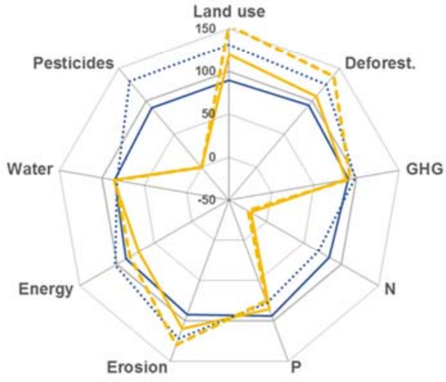

50% reduction in FCF

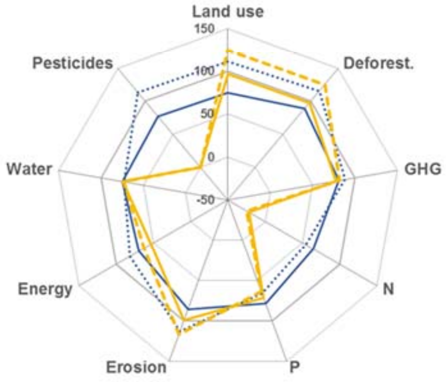

100% reduction in FCF

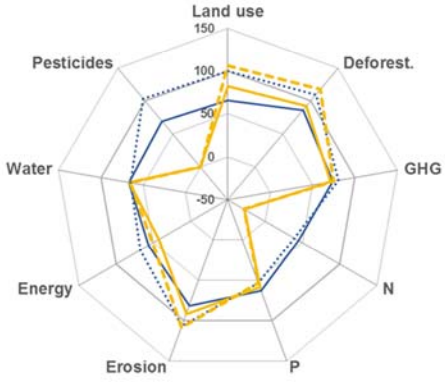

The numbers on the axis indicate % impact, rel. to the reference scenario (which is represented by the thicker grey line at 100%)

Supplementary Figure 10: Year 2050 relative environmental impacts of a full conversion to organic agriculture in combination with complementary food systems strategies under high yield gaps

Percentage of environmental impacts with respect to the reference scenario (i.e. 0% organic agriculture, no changes in livestock feed and food waste; dark grey line); Calories are kept constant for all scenarios without food wastage reduction; high yield gaps[10] assumed; the lines show the range of impacts for 0% (dark blue) to 100% organic shares (yellow) under the respective reduction of food-competing feed and wastage as indicated for each panel; the solid lines indicate environmental impacts without impacts of climate change on yields, the dotted lines indicate environmental impacts with ICC. The thicker solid grey gridline indicates the 100% level, i.e. the reference scenario. Food-competing feed (FCF) use is at the levels of the reference scenario on the left (i.e. 0% reduction in FCF) and changes towards zero FCF use to the right (i.e. 100% reduction in FCF); wastage reduction changes from 0% (top panel) to 50% (bottom). Indicators displayed: cropland use, deforestation, GHG emissions (incl. deforestation, organic soils), N- and P-surplus, water use, non-renewable energy use, soil erosion, pesticide use.

0% food wastage reduction

0% reduction in food-competing feed (FCF)	50% reduction in FCF	100% reduction in FCF

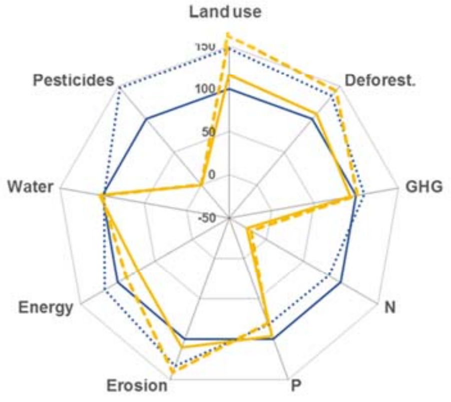

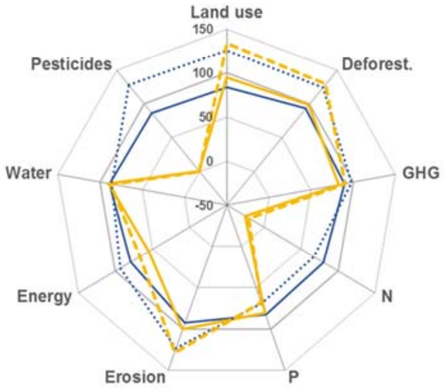

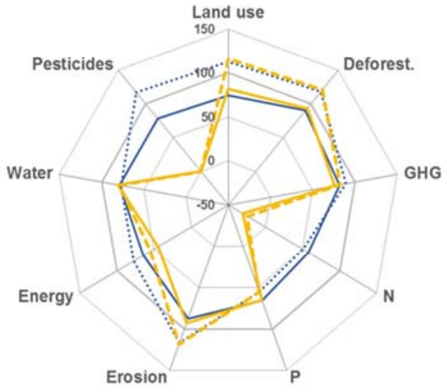

The numbers on the axis indicate % impact, rel. to the reference scenario (which is represented by the thicker grey line at 100%)

25% food wastage reduction

0% reduction in food-competing feed (FCF)	50% reduction in FCF	100% reduction in FCF

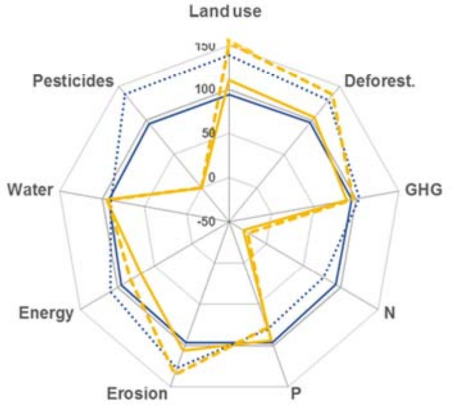

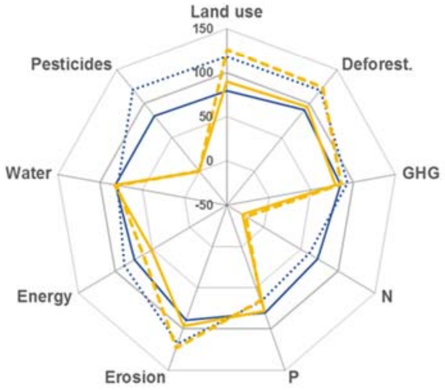

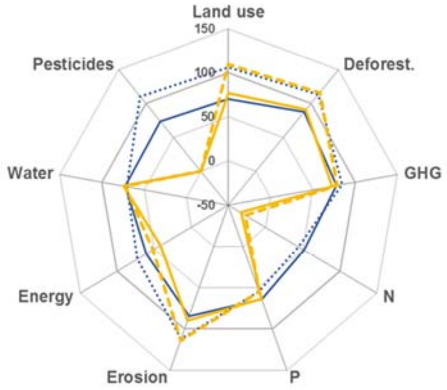

The numbers on the axis indicate % impact, rel. to the reference scenario (which is represented by the thicker grey line at 100%)

50% food wastage reduction

0% reduction in food-competing feed (FCF)	50% reduction in FCF	100% reduction in FCF

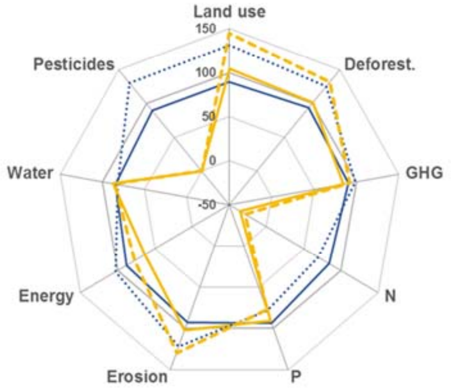

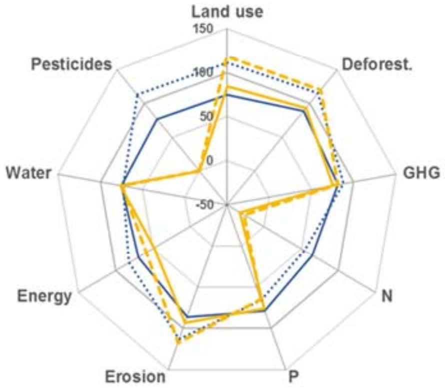

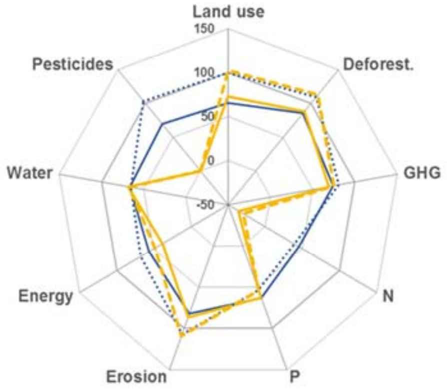

The numbers on the axis indicate % impact, rel. to the reference scenario (which is represented by the thicker grey line at 100%)

Supplementary Figure 11: Year 2050 relative environmental impacts of a full conversion to organic agriculture in combination with complementary food systems strategies under low yield gaps

Percentage of environmental impacts with respect to the reference scenario (i.e. 0% organic agriculture, no changes in livestock feed and food waste; dark grey line); Calories are kept constant for all scenarios without food wastage reduction; low yield gaps[9] assumed; the lines show the range of impacts for 0% (dark blue) to 100% organic shares (yellow) under the respective reduction of food-competing feed and wastage as indicated for each panel; the solid lines indicate environmental impacts without impacts of climate change on yields, the dotted lines indicate environmental impacts with impacts of CC. The thicker solid grey gridline indicates the 100% level, i.e. the reference scenario. Food-competing feed (FCF) use is at the levels of the reference scenario on the left (i.e. 0% reduction in FCF) and changes towards zero FCF use to the right (i.e. 100% reduction in FCF); wastage reduction is displayed in the three panels, from 0% (top) to 50% (bottom).The indicators are cropland use, deforestation, GHG emissions (incl. deforestation and organic soils), N- and P-surplus, water use, non-renewable energy use, soil erosion and pesticide use.

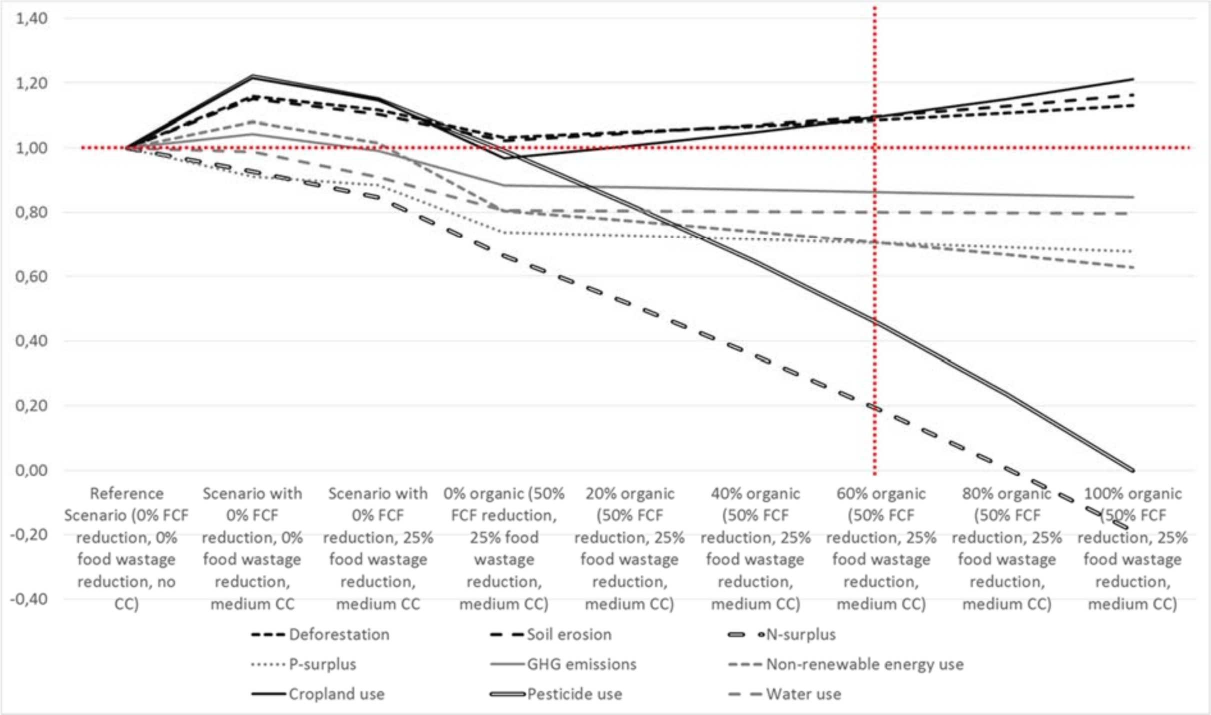

Supplementary Figure 12: Relative change of environmental impacts with respect to the reference scenario for increasing shares of organic production. The dotted red horizontal line indicates the level for the reference scenario, the dotted red vertical line indicates the values for 60% conversion to organic agriculture. Calories are kept constant for all scenarios without food wastage reduction; high yield gaps, 50% food-competing feed (FCF) reduction, 25% food wastage reduction and intermediate impact of CC assumed; to the left, for comparison, the scenarios without food-competing feed and food wastage reduction but CC impact, as well as no food-competing feed reduction, 25% food wastage reduction and CC impact are displayed.

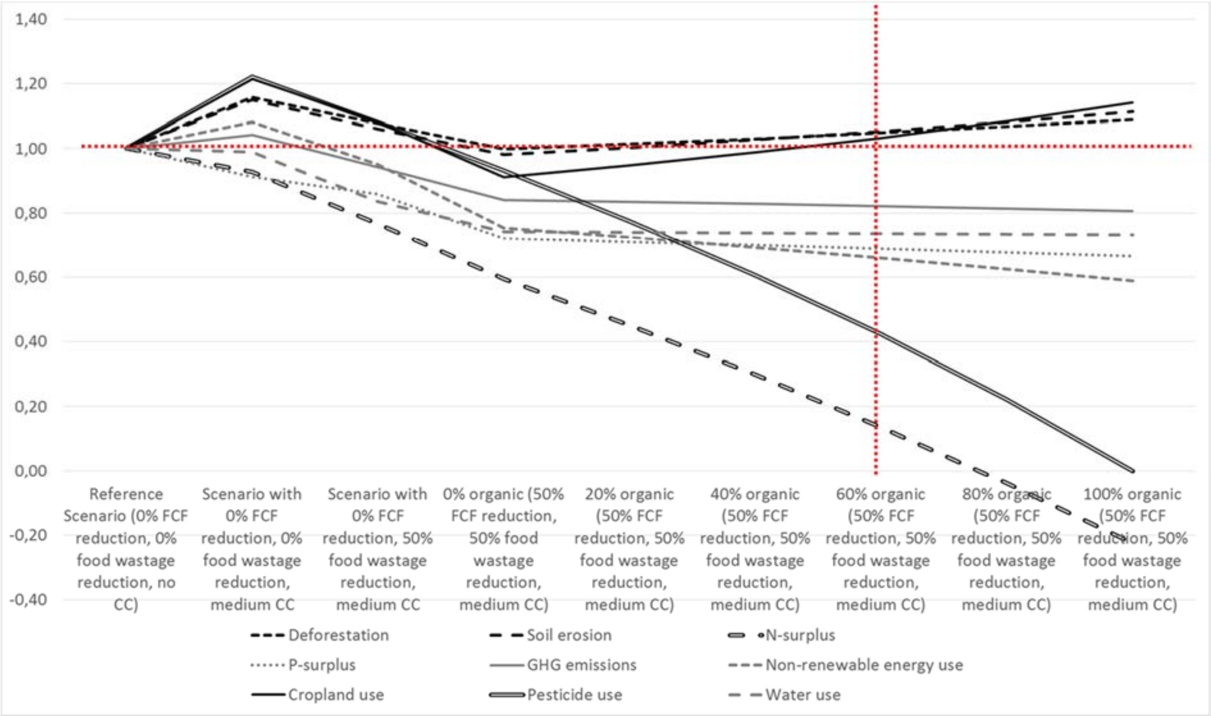

Supplementary Figure 13: Relative change of environmental impacts with respect to the reference scenario for increasing shares of organic production. The dotted red horizontal line indicates the level for the reference scenario, the dotted red vertical line indicates the values for 60% conversion to organic agriculture. Calories are kept constant for all scenarios without food wastage reduction; high yield gaps, 50% food-competing feed (FCF) reduction, 50% food wastage reduction and intermediate impact of CC assumed; to the left, for comparison, the scenarios without food-competing feed and food wastage reduction but CC impact, as well as no food-competing feed reduction, 50% food wastage reduction and CC impact are displayed.

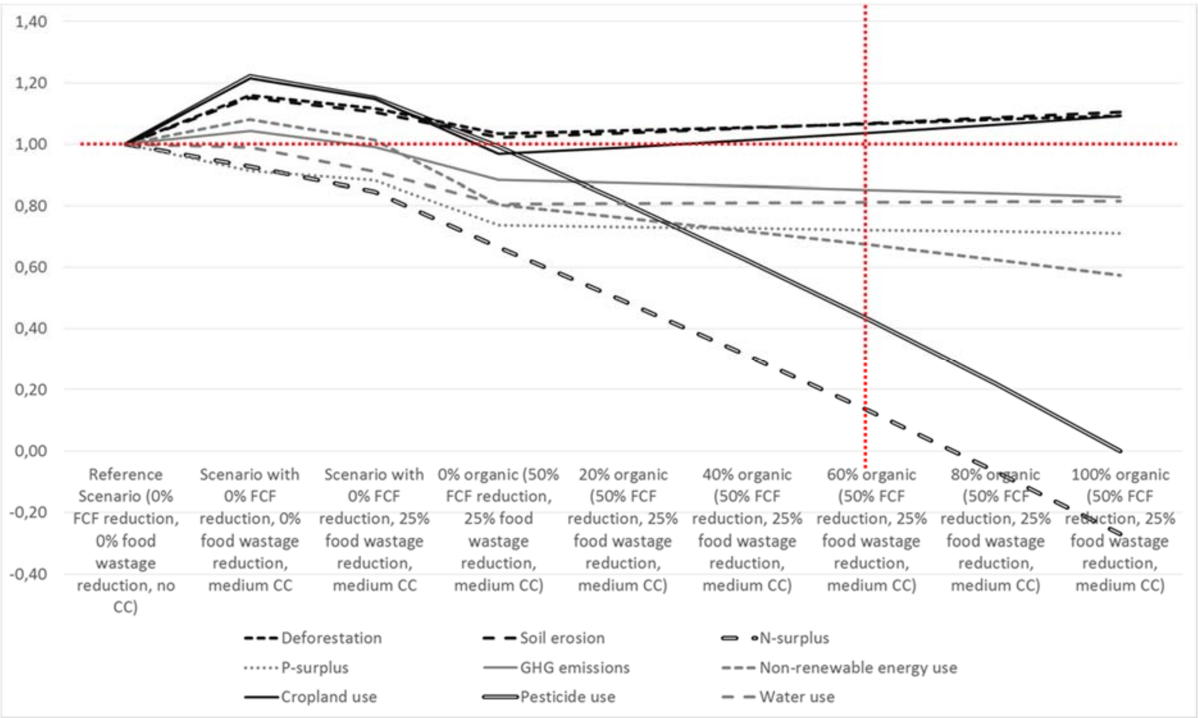

Supplementary Figure 14: Relative change of environmental impacts with respect to the reference scenario for increasing shares of organic production. The dotted red horizontal line indicates the level for the reference scenario, the dotted red vertical line indicates the values for 60% conversion to organic agriculture. Calories are kept constant for all scenarios without food wastage reduction; low yield gaps, 50% food-competing feed (FCF) reduction, 25% food wastage reduction and intermediate impact of CC assumed; to the left, for comparison, the scenarios without food-competing feed and food wastage reduction but CC impact, as well as no food-competing feed reduction, 25% food wastage reduction and CC impact are displayed.

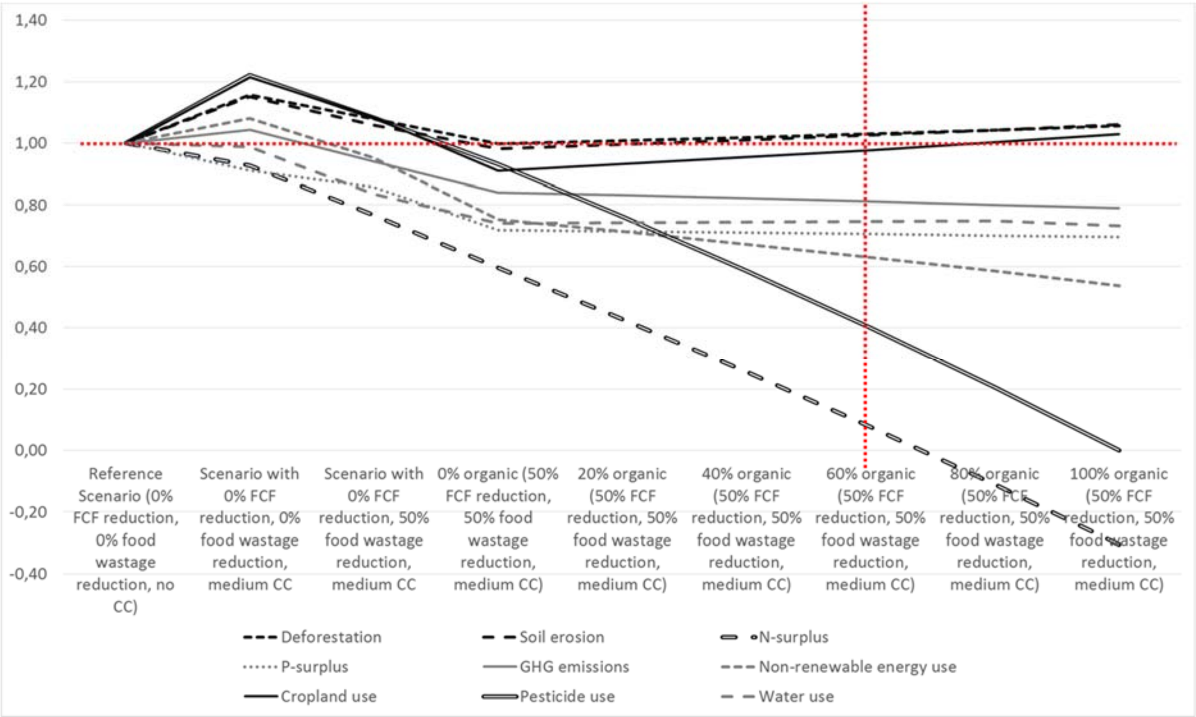

Supplementary Figure 15: Relative change of environmental impacts with respect to the reference scenario for increasing shares of organic production. The dotted red horizontal line indicates the level for the reference scenario, the dotted red vertical line indicates the values for 60% conversion to organic agriculture. Calories are kept constant for all scenarios without food wastage reduction; low yield gaps, 50% food-competing feed (FCF) reduction, 50% food wastage reduction and intermediate impact of CC assumed; to the left, for comparison, the scenarios without food-competing feed and food wastage reduction but CC impact, as well as no food-competing feed reduction, 50% food wastage reduction and CC impact are displayed.
